# Investigating the Effects of Conditioned Media from Stem Cells of Human Exfoliated Deciduous Teeth on Dental Pulp Stem Cells

**DOI:** 10.3390/biomedicines10040906

**Published:** 2022-04-15

**Authors:** Huong Thu Vu, Mi-Ran Han, Jun-Haeng Lee, Jong-Soo Kim, Ji-Sun Shin, Ji-Young Yoon, Jeong-Hui Park, Khandmaa Dashnyam, Jonathan Campbell Knowles, Hae-Hyoung Lee, Jong-Bin Kim, Jung-Hwan Lee

**Affiliations:** 1 Department of Pediatric Dentistry, College of Dentistry, Dankook University, 119 Dandae-ro, Cheonan 31116, Korea; huong.vuthudr@gmail.com (H.T.V.); miraneee@dankook.ac.kr (M.-R.H.); haeng119@naver.com (J.-H.L.); jskim@dku.edu (J.-S.K.); pedosin@dankook.ac.kr (J.-S.S.); 2 Institute of Tissue Regeneration Engineering (ITREN), Dankook University, 119 Dandae-ro, Cheonan 31116, Korea; wisdom7970@gmail.com (J.-Y.Y.); shurins@naver.com (J.-H.P.); khandmaa@naver.com (K.D.); j.knowles@eastman.ucl.ac.uk (J.C.K.); haelee@dku.edu (H.-H.L.); 3 Department of Biomaterials science, College of Dentistry, Dankook University, 119 Dandae-ro, Cheonan 31116, Korea; 4 Department of Nanobiomedical Science & BK21 PLUS NBM Global Research Center for Regenerative Medicine, Dankook University, 119 Dandae-ro, Cheonan 31116, Korea; 5 UCL Eastman-Korea Dental Medicine Innovation Centre, Dankook University, 119 Dandae-ro, Cheonan 31116, Korea; 6 Mechanobiology Dental Medicine Research Centre, Cheonan 31116, Korea; 7 Cell & Matter Institue, Dankook University, Cheonan 31116, Korea; 8 Division of Biomaterials and Tissue Engineering, Eastman Dental Institute, Royal Free Hospital, Rowland Hill Street, London NW3 2PF, UK; 9 The Discoveries Centre for Regenerative and Precision Medicine, Eastman Dental Institute, University College, London WC1E 6BT, UK; 10 Drug Research Institute, Mongolian Pharmaceutical University & Monos Group, Ulaanbaatar 14250, Mongolia

**Keywords:** conditioned medium, dental pulp stem cell, human exfoliated deciduous teeth, cytokines, odontoblast/osteogenic differentiation

## Abstract

Pulp regeneration has recently attracted interest in modern dentistry. However, the success ratio of pulp regeneration is low due to the compromising potential of stem cells, such as their survival, migration, and odontoblastic differentiation. Stem cells from human exfoliated deciduous teeth (SHED) have been considered a promising tool for regenerative therapy due to their ability to secrete multiple factors that are essential for tissue regeneration, which is achieved by minimally invasive procedures with fewer ethical or legal concerns than those of other procedures. The aim of this study is to investigate the potency of SHED-derived conditioned media (SHED CM) on dental pulp stem cells (DPSCs), a major type of mesenchymal stem cells for dental pulp regeneration. Our results show the promotive efficiency of SHED CM on the proliferation, survival rate, and migration of DPSCs in a dose-dependent manner. Upregulation of odontoblast/osteogenic-related marker genes, such as ALP, DSPP, DMP1, OCN, and RUNX2, and enhanced mineral deposition of impaired DPSCs are also observed in the presence of SHED CM. The analysis of SHED CM found that a variety of cytokines and growth factors have positive effects on cell proliferation, migration, anti-apoptosis, and odontoblast/osteogenic differentiation. These findings suggest that SHED CM could provide some benefits to DPSCs in pulp regeneration.

## 1. Introduction

The continuously increasing progress in stem cell biology offers promising therapies to repair damaged or missing dental tissues. Tissue regeneration emerged in modern endodontics when numerous mesenchymal stem cells (MSCs) were reported to regenerate pulp-like tissue, such as permanent tooth-isolated dental pulp stem cells (DPSCs), stem cells isolated from the human pulp of exfoliated deciduous teeth (SHED), periodontal ligament stem cells, stem cells from the apical papilla (SCAP), dental follicle cells, or bone marrow stem cells (BMSCs) [1,2,3]. In current root canal treatment modalities, the process of root canal preparation can weaken the integrity of the tooth structure and make them more susceptible to fracture [4,5]. Despite advances in biocompatible materials, there is a considerable concern of tooth discoloration after root canal treatments, and this discoloration impairs the aesthetic outcome of the treated tooth [6,7]. Therefore, the idea of pulp regeneration was proposed, and advanced knowledge of stem cells and biotechnology has aided in regenerating the stem cell-based pulp-dentin complex. Several considerable efforts have been made, and certain achievements have been made both in vitro and in vivo [8,9,10]. Through transplanting autologous dental pulp stem cells, clinicians recently demonstrated that pulp regeneration is no longer an unachievable goal with compromising results [11,12,13,14].

Permanent tooth-derived pulp dental stem cells (DPSCs), which are one of the main sources of stem cells for pulp regenerative therapy, are easily obtained from unnecessary teeth, such as third molars or premolars extracted teeth, due to orthodontic demand. Many studies have demonstrated the ability of DPSCs to differentiate into odontoblasts and neurons as well as support vascular network formation, which are the basic components of a dentin/pulp-like complex [2,11,15]. However, the DPSC capacity in pulp regeneration showed an age-related decline, partly because of the reduction in cell survival, proliferation, and migration and the decrease in stem cell properties in aged DPSCs [16,17,18,19]. Changes in the microenvironment influence cell fate in terms of adhesion, migration, and differentiation; therefore, researchers have made more intensive efforts to engineer optimal microenvironments that enhance the survival, proliferation, and differentiation of DPSCs and attract neighboring progenitor cells that migrate to prepared root canals [20,21,22].

**Figure 1:**
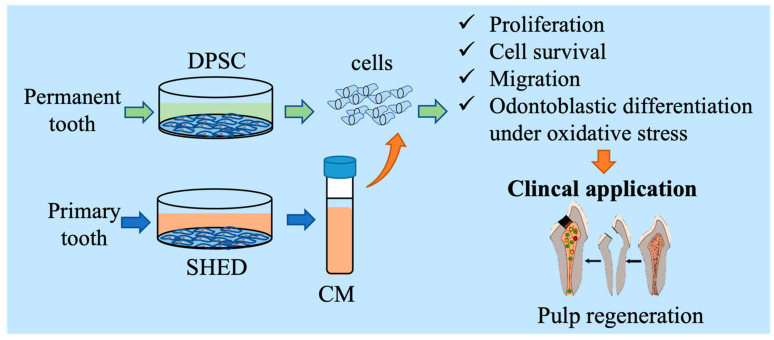
Graphic abstract. SHED were collected from the pulp tissue of primary teeth, and DPSC were collected from the pulp tissue of permanent teeth. The cytokines/growth factors contained in SHED-cultured conditioned media promoted DPSC proliferation, survival, migration, and cell differentiation in pulp regenerative therapy.

In recent decades, MSC secretomes, which include the conditioned medium (CM) and extracellular vesicle fraction, have been proposed for use in regenerative medicine and tissue engineering due to their bioactive function and regenerative ability [23,24,25]. The different sources of stem cells express distinctive cytokine profiles and properties, and their released secretomes may have different potentials in tissue regeneration. BMSC-derived CM enhanced cell migration and osteo/odontogenic differentiation potential, but it inhibited the proliferation of DPSCs [26,27]. In a comparison study, CM obtained from SCAPs had a greater effect on migration and osteo/odontogenic and neurogenic effects on DPSCs than that of CM from BMSCs; however, SCAP CM had no effect on cell proliferation [27]. Compared to other sources of dental stem cells, SHED are considered a promising tool due to their excellent potential for osteogenic, angiogenic, and neurotrophic regeneration with fewer ethical or legal concerns. SHED were more efficient in secreting osteogenic differentiation-related growth factors, while DPSCs from aged permanent teeth secreted higher levels of proinflammatory cytokines [28]. A recent study revealed that cytokines involved in immunomodulation, proliferation, odontogenesis, and osteogenesis were stronger in SHED CM [29]. These effects of SHED CM are associated with multiple growth factors, such as OPG, OPN, BMP-2, BMP-4, TGF-β, M-CSF, MCP-1, ANG, bFGF, VEGF-C, and VEGF-A, which are bone metabolism-related factors and angiogenesis markers [29,30,31]. It was also found to contain BDNF, beta-NGF, GDNF, and NT-3, members of the neurotrophic family [31]. Several studies revealed that SHED CM had a protective function through its anti-inflammatory cytokine angiogenic and anti-apoptosis factors [30,32,33]. Cytokines and growth factors contained in SHED CM also induced the migration of adjacent or distant cells to the target position [33]. Because of these properties, SHED CM is expected to be a potential tool in tissue regenerative therapy. To our knowledge, this is the first study to investigate the effect of SHED CM on DPSC. In this study, with the hypothesis that factors containing SHED CM would be capable of enhancing the pulp-dentin-like complex, we evaluated the influence of SHED CM on DPSCs in vivo for proliferation, migration, cell death, and odontoblast-like cell differentiation (Figure 1).

## 2. Materials and Methods

### 2.1. Primary Culture and Expansion of Dental Pulp Stem Cell

The following two cell types were used in this study: SHED was collected from the exfoliated deciduous tooth (6 years old, male), and DPSC were isolated from the third molar (18 years old, male). These teeth were extracted for clinical purposes at the Dental Hospital, Dankook University, under guidelines approved by the Ethical Committee of the Institutional Review Board of Dankook University Dental Hospital (IRB number DKUDH 2019-10-001). Informed consent was obtained from all participants.

The extracted teeth were soaked in 5 mL Hanks’ balanced salt solution (HBSS, Welgene, Gyeongsangbuk, Korea) containing 1% penicillin–streptomycin (PS, Gibco, Thermo Fisher, Waltham, MA, USA) at 4 °C. Pulp tissue was minced and immersed in a solution of 2 mg/mL collagenase type I (Worthington Biochemical, Lakewood, NJ, USA) and 4 mg/mL Dipase II (Sigma-Aldrich, St. Louis, MO, USA) for 1 h at 37 °C in a water bath. The dissociated cell suspension was suspended in 1 mL growth medium (αMEM, MEM Alpha Modification, with L-glutamine, with Ribo- and deoxyribonucleosides, HyClone, Logan, Utah, USA) supplemented with 15% fetal bovine serum (FBS, Corning, Corning, NY, USA), 2 mM L-glutamine (Thermo Fisher), and 0.1 mM L-ascorbic acid phosphate (Thermo Fisher) and then centrifuged at 1500 rpm for 5 min at room temperature. After removing the supernatant, 1 mL growth medium was added to the cell pellet, and this single-cell suspension was filtered with 75 μm trainer (Falcon, Corning) before being plated on culture dishes (60 mm × 15 mm, Falcon, Corning) and incubated at 37 °C in an atmosphere containing 5% CO_2_ at 95% humidity. The culture medium was changed after 48 h of initial incubation and then every 7 days thereafter. After 10 to 14 days, single-cell-derived colonies were collected and subcultured. Then, the cells were digested with 0.25% EDTA-Trypsin (Thermo Fisher) and passaged when the cell confluence reached 80–90%. Cells from passages 4 to 10 were used for further experiments. Isolation and adhesion of human dental pulp stem cells (hDPSCs)

### 2.2. Preparation of Conditioned Medium

Before starting the preparation of SHED-CM, we confirmed the multiple differentiation potentials of isolated SHED (Supplementary Figure S1).

SHED at the 5th passage achieved 70%–80% confluence in 100 mm cell culture dishes (uncoated, SPL Life Sciences, Gyeonggi, Korea), were washed thoroughly 3 times with PBS 1X (Tech & Innovation, Gangwon, Korea) and were then replenished with serum-free αMEM. After 48 h of incubation at 37 °C in 5% CO_2_, the conditioned medium (CM) was collected and centrifuged at 3000 rpm for 5 min at 4 °C to remove cell debris, and then the supernatant was passed through 0.2 μm filters to obtain the final CM. Control conditioned media was collected from serum-free αMEM (SFM) and incubated for 48 h under the same conditions as the conditioned medium. The number of live cells was also calculated to ensure a cell viability higher than 90%. All CM and SFM were subpackaged in 2 mL tubes and stored at −80 °C until use in subsequent experiments. (Supplementary Figure S2).

### 2.3. Proliferation Assays

To measure the effect of SHED CM on the viability and proliferation of DPSCs, 100 μL of cell suspension (αMEM, 10% FBS) was plated into 96-well plates (SPL Life Sciences) at a density of 5 × 10^3^ cells/well, and overnight incubation allowed for cell adhesion. Cells were replenished with CM, 50% CM (half of CM and half of SFM) or SFM followed by 1X PBS washing to completely remove the remaining FBS. Following incubation for 24 h, 10μL CCK-8 (Dojindo, Kumamoto, Japan) was added to each well of the plate and incubated for 2 h at 37 °C. The absorbance at 450 nm was recorded using a microplate reader (Thermo Fisher VarioskanTM LUX, Waltham, MA, USA). Cell viability (%) was quantified by the average absorbance of the CM, 50% CM treated group/average absorbance of the SFM group × 100%. Cell survival was also examined by Live and Dead staining (0.5 µM calcein AM and 2 µM ethidium homodimer-1 solutions, Thermo Fisher, Waltham, MA, USA), and images were taken using an optical microscope (IX71, Olympus, Tokyo, Japan).

### 2.4. Cell migration Assay

**Figure 2:**
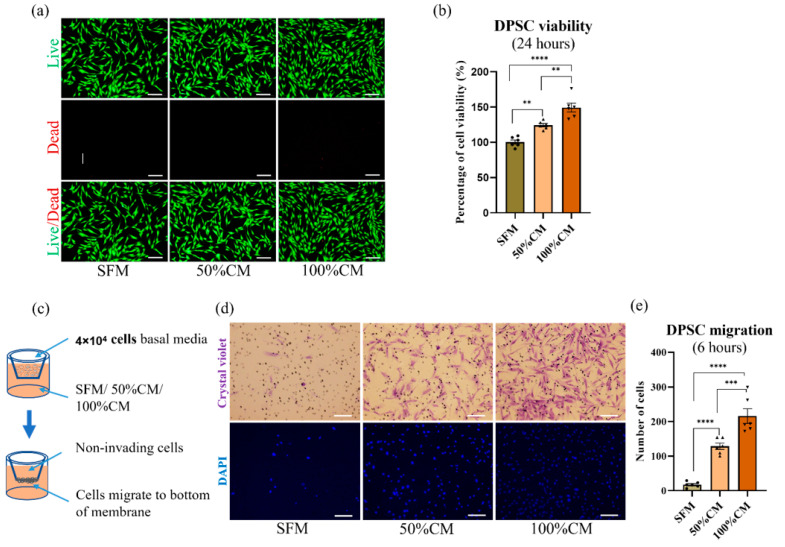
Effect of stem cells isolated from human pulp of exfoliated deciduous tooth-derived conditioned media (CM) on dental pulp stem cells (DPSCs) regarding proliferation and migration. (**a**) Presentative live and dead images of SHED 24 h after treatment with 100% CM versus 50% CM and SFM. Scale bar represents 200 µm. (**b**) The effect of SHED CM after treatment for 24 h on the proliferation of SHED was quantified by CCK-8 assay. *n* = 6. (**c**) Schematic of migration assays. A total of 4 × 10^4^ cells in 100 µL were seeded in the upper compartment, while the lower compartment contained 350 µL 100% CM, 50% CM or SFM. (**d**) Representative images of migrated SHED in the lower compartment of membrane after 6 h of treatment. (**e**) Cells counted in the lower compartment in 6 random fields. All data are represented as the mean ± standard deviation (SD). The statistical significance was calculated using one-way analysis of variance (ANOVA) to compare groups. Represents ** *p* < 0.01, *** *p* < 0.001, **** *p* < 0.0001, scale bar = 200 µm.

The chemical attraction effect of SHED CM on DPSCs was evaluated by a transwell migration chamber assay. Briefly, 4 × 10^4^ DPSCs in 100 μL serum-free αMEM were plated on top of the filter membrane of a transwell insert (8 μm pore size, Corning). Three kinds of culture media (350 μL), 100% SHED CM, 50% SHED CM, or SFM, were added to the lower chamber (24-well plate) (Figure 2c). After 6 h of incubation at 37 °C, 5% CO_2_, the unmigrated DPSCs on the upper surface were removed, and the migrated cells on the lower surface were stained with a crystal violet solution (Sigma–Aldrich) and DAPI (4’,6-diamidino-2-phenylindole, Thermo Fisher). The stained cells were observed under an inverted microscope (IX71, Olympus, Tokyo, Japan) at × 100 magnification.

### 2.5. Antioxidative Stress

To mimic the microenvironment of oxidative stress detected at the transplantation site, hydrogen peroxide (H_2_O_2,_ Sigma–Aldrich) is widely used as an inducer of oxidative stress. At low levels, H_2_O_2_ has a beneficial role in basic cellular activity such as proliferation and migration. High levels of H_2_O_2_ can cause oxidative stress and lead to adverse effects on cell activity. A working concentration of H_2_O_2_ that killed approximately 50% of DPSCs after 12 h of incubation was used to test the hypothesis that SHED CM could have an antioxidant property to protect DPSCs against H_2_O_2_-induced cellular damage. DPSCs (5 × 10^3^ cells/well) plated in 96-well plates were treated with different concentrations of H_2_O_2_ (0, 100, 200, 400 µM) in serum-free αMEM. The protective function of CM on cell apoptosis was evaluated by CCK-8 and live and dead assays as described above. Cells without H_2_O_2_ treatment were chosen as the control group.

### 2.6. Osteogenic Differentiation Assays

The impaired effect of H_2_O_2_ on the differentiation potential of DPSCs was first investigated. The density of 1 × 10^4^ DPSCs was seeded on a 24-well plate overnight and treated with different concentrations of H_2_O_2_ (50 μM to 200 μM) for 24 h, changing to osteogenic differentiation media (OM) (αMEM, 10% FBS, 1% PS, 10 mM transforming growth factor-β1 (TGF-β1, Peprotech), 100 nM dexamethasone (Sigma), and 50 μg/mL ascorbic acid) at 37 °C in 5% CO_2_. Osteogenic differentiation capacity was determined by ARS staining after 21 days.

To evaluate the effect of SHED CM on DPSCs under oxidative stress conditions, DPSCs were treated with H_2_O_2_ at a concentration of 25 μM for 24 h before osteogenic induction. DPSCs that were not exposed to H2O2 were used as the control group. Differentiation efficiency was monitored by alkaline phosphatase (*ALP*) and Alizarin Red S staining (ARS). ALP staining was performed on Days 3 and 7 by using a staining kit (SIGMAFAST^TM^ BCIP^®^/NBT tablet, Sigma–Aldrich). On Days 3 and 7, the differentiated cells were fixed with 4% paraformaldehyde (Tech & Innovation) at 37 °C for 15 min and then stained with ALP solution dissolved in distilled water for 1 h at 37 °C in the dark. The cells were stained with 40 mM Alizarin red S reagent (pH = 4.2) (Sigma–Aldrich) on Days 21 and 28. After staining and washing five times with distilled water, optical images of the ALP activity and ARS staining were taken via light microscopy (Olympus IX71, Shinjuku, Tokyo, Japan). For ARS staining quantification, the stained calcium deposits were dissolved in 10% (weight/volume) cetylpyridinium chloride (CPC, Sigma–Aldrich) solution on a rocking shaker for 30 min at room temperature. ARS extracts were aliquoted into 96-well plates (100 µL/well) and read at 562 nm.

### 2.7. Cytokine Profiling

To identify factors responsible for the therapeutic effects of SHED CM, the cytokines and related biomarkers were profiled. SHED CM or SFM was collected by the described method and stored at −80 °C to maintain the biological properties before cytokine antibody analysis. The Fullmoonbio Cytokine Profiling antibody array (cat. SCK100/SEV07-SCK10, Full Moon BioSystems, Sunnyvale, CA, USA) contains two identical array slides featuring 310 antibodies for profiling cytokines, and related biomarkers human cells, tissues, serum, or culture media were used. The cytokines with fold changes > 1 (*p* < 0.05) were used for integrative analyses, and data were analyzed using ExDEGA v.3.2.1 software (eBiogen, Seoul, Korea). Figure 4a briefly describes the process of cytokine analysis from cell culture to the detection of cytokines and growth factors contained in SHED CM and SFM.

### 2.8. Quantitative Real Time Polymerase Chain Reaction (qRT–PCR)

DPSCs were differentiated into 2 groups of media (CM and SFM) for 3 and 7 days. Odontoblast differentiation-related gene expression levels of alkaline phosphatase (*ALP*), dentin sialophosphoprotein (DSPP), dentin matrix protein 1 (*DMP1*), osteocalcin (*OCN*), and runt-related transcription factor 2 (RUNX2), which are relevant to the odontogenic differentiation of cells, were measured. Glyceraldehyde-3-phosphate dehydrogenase (*GAPDH*) serves as the housekeeping gene. Undifferentiated DPSCs were used as a control throughout the study. The results are expressed as mRNA relative expression.

### 2.9. Statistical Analyses

Data are expressed as the mean ± standard deviation, and statistical analyses were performed using GraphPad Prism 8 software (San Diego, CA, USA). Statistical significance between groups was evaluated by one-way analysis of variance (ANOVA) followed by Dunnett’s multiple comparison tests, and *p* < 0.05 was considered significant.

## 3. Results

### 3.1. Characteristics of DPSCs and SHEDs

Primary dental stem cell adherence could be observed on the culture plate wall 16–24 h after seeding, and the formation of cell colonies was observed after 4 or 6 days. Most cells had a typical fibroblast-like appearance. The cell morphology was polygonal, spindle-shaped, or oval-shaped, consistent with known MSC phenotypic characteristics. The isolated SHED showed mineral depostition (ARS) and neutral lipid accumulation (Oil Red Staining) under the proper differentiated culture conditions, thus confirming the differentiation capacity into osteogenic and adipogenic lineages (Supplementary Figure S1).

### 3.2. Effect of CM on Cell Viability and Proliferation

To compare the effects of CM and SFM on cell proliferation, CCK-8 assays were performed at different time points and after 24 h and 48 h of treatment. As shown in Figure 2a,b and Supplementary Figure S3 SHED CM significantly promoted the proliferation of DPSCs, and this effect strengthened with increasing CM concentrations (*p* < 0.0001). More significant growth promotion was observed in 100% SHED CM-treated cultures, with promotion up by 50% (149.3 ± 15.27) and 30% in 50% CM (124.4 ± 5.64) compared to that of SFM after 24 h. This effect continued after 48 h of incubation, by a significant of 61.73 ± 8.58 and 35.47 ± 8.58% in whole and 50% SHED CM, respectively. The live and dead assay showed consistently with CCK-8 assay result, the highest level of cell proliferation in 100% CM, followed by 50% CM, and lowest in SFM.

### 3.3. SHED CM Attracted DPSC Migration

CM-treated DPSCs showed a remarkably higher cell migration rate than that of cells treated with SFM (*p* *<* 0.001). Quantitative analysis showed a strong promotive effect of CM on cell migration proportional to the CM concentration used. One hundred percent CM induced the greatest migration, with an average of 216 ± 52 cells, and 50% CM was less efficient than CM, with 128 ± 22 cells. SFM seemed to not attract cell migration when few cells migrated to the bottom surface of the membrane (17 ± 8 cells) (Figure 2d,e).

### 3.4. Protective Effect of SHED CM against H_2_O_2_-Induced Cell Death on DPSCs

**Figure 3:**
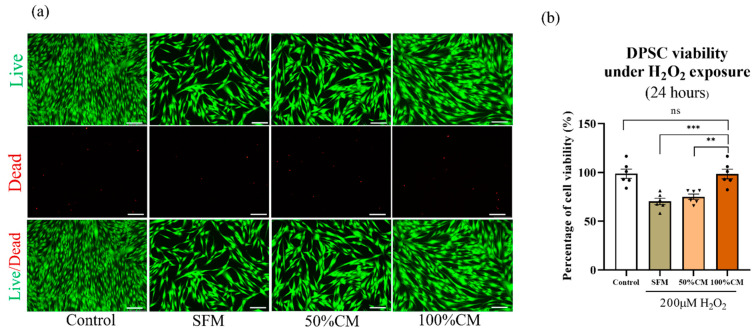
Treatment with SHED CM improved the viability of DPSC after H_2_O_2_ exposure. DPSCs were cultured with 200 µM H_2_O_2_ for 12 h before shifting to 100% CM, 50% CM and SFM for another 12 h. (**a**) Representative live and dead images of DPSCs confirmed the antioxidative stress effect of SHED-CM. (**b**) Cell viability was determined by CCK-8 analysis. All data are represented as the mean ± SD. The statistical significance was calculated using one-way analysis of variance (ANOVA) to compare groups. Represents ** *p* < 0.01, *** *p* < 0.001, ns = not significant, scale bar = 200 µm.

We first evaluated the toxicity of H_2_O_2_ on the proliferation of DPSCs to select the dose for subsequent experiments. Concentrations of 100, 200, 300, and 400 μM of H_2_O_2_ were used to treat DPSCs for 12 h. Based on the CCK-8 assay, a concentration of 200 μM was chosen because the cell viability was significantly decreased by approximately 50% after 12 h of H_2_O_2_ treatment (Supplementary Figure S4a). As shown in Figure 3, posttreatment with CM for 12 h reversed the H_2_O_2_-induced damage and significantly decreased cell death under 200 μM H_2_O_2_-induced oxidative stress conditions. The concentration of 100% CM was more efficient than 50% CM, and cell viability increased by 28.09 ± 4.64 in the CM group and 4.78 ± 4.64 in the 50% CM group compared to the SFM group (Figure 3b). In the same way, the live and dead assays confirmed the protective effect of 100% CM on DPSCs from H_2_O_2_ (Figure 3a). These results suggest that CM could contain proteins or mRNAs that protect cells from H_2_O_2_-induced apoptosis.

### 3.5. Cytokine Profiling Results

**Figure 4:**
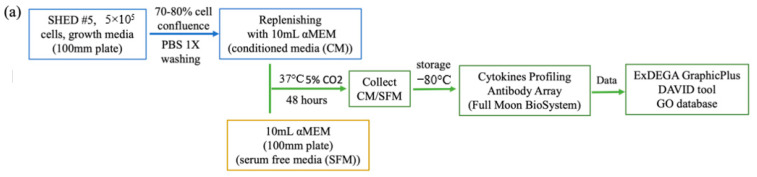

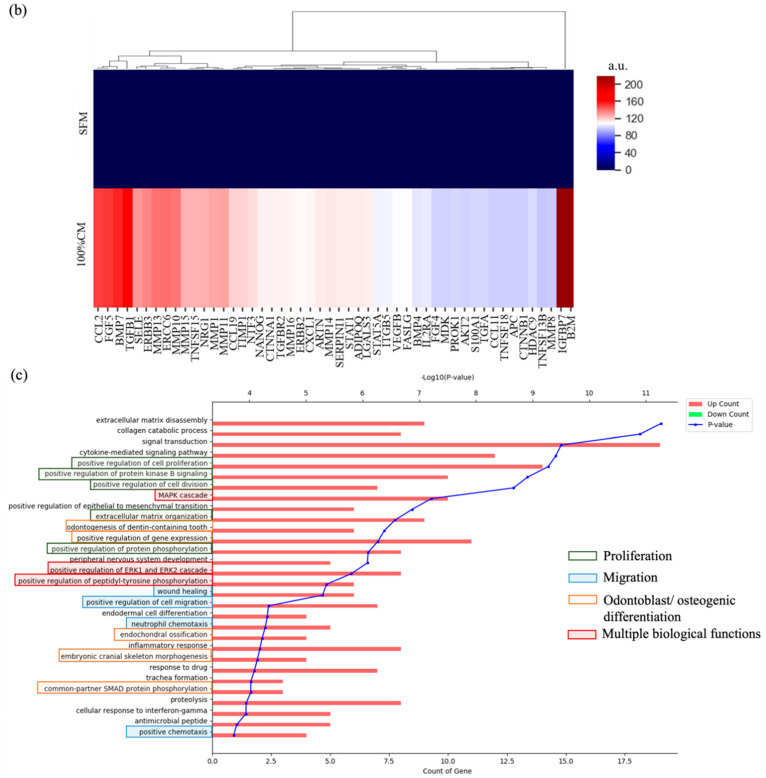
Cytokine profiling array. (**a**) The process of cytokine analysis is briefly described. SHED at the 5th passage achieved 70%–80% confluence and was replenished with serum-free αMEM. After 48 h, the conditioned medium (CM) was collected. Control conditioned media was collected from serum-free αMEM (SFM) under the same conditions as conditioned medium. All CM and SFM were stored at −80 °C to maintain the biological properties before cytokine antibody analysis. The Fullmoonbio Cytokine Profiling antibody array features 310 antibodies for profiling cytokines, and related biomarkers were used. The data were analyzed with the ExDEGA GraphicPlus and DAVID tools based on the Gene Ontology (GO) database. (**b**) A clustering heatmap based on the 50 top cytokines showed a significant difference between SFM and 100% CM. (**c**) Using the 50 top cytokine lists, the DAVID v6.8 analysis tool with the Gene Ontology Term Biological Process database and ExDEGA Graphic Plus were used to build a functional annotation chart. The graph shows that SHED CM containing growth factors and cytokines has a positive effect on cell proliferation, migration, survival, and osteogenic differentiation. Pathways/biological activities related to upregulation of proliferation, migration, odontoblast/osteogenic differentiation, and multiple biological functions (proliferation, migration, downregulation of cell apoptosis, differentiation including downregulation of cell apoptosis) are marked in different colored boxes (green, blue, orange, and red, respectively).

The data are expressed as the fold change value/arbitrary value in the ExDEGA report Excel file. The Gene Ontology (GO) database and ExDEGA GraphicPlus were used for further analysis. Functional annotation analysis using a DAVID tool was used to predict the major functions of cytokines by statistical analysis of their correlation based on various databases. Of the 310 cytokines, 50 showed the highest values in SHED CM compared to that of SFM. A clustering heatmap based on the 50 top cytokines provided a significant difference between SFM and 100% CM (Figure 4b). These cytokine lists were put into the DAVID v6.8 analysis tool, and all listed cytokines were involved in functionally identified genes in the database. Cytokines involved in biological functions by Gene Ontology Term BP are described in Supplementary Table S1. This result indicates that growth factors and cytokines found in SHED CM have positive effects on cell proliferation, migration, survival, and osteogenic differentiation, such as transforming growth factor beta receptor (TGF-β), bone morphogenetic protein (BMP), fibroblast growth factors (FGF), vascular endothelial growth factor (VEGF), matrix metalloproteinase (MMP), and the families of interleukin (IL). The results from ExDEGA Graphic analysis were consistent with the in vitro study when SHED CM contained cytokines associated with the upregulation of cell proliferation, modulation of cell apoptosis, cell migration, and enhanced odontoblast/osteogenic differentiation. The SHED CM secretomes also evolved in different biological processes and signaling pathways, such as the MAPK pathway and SMAD protein phosphorylation, which are considered to play important roles in intracellular biological activities (Figure 4c).

### 3.6. Odontoblast/Osteoblast Induction

After 24 h of exposure to 25 μm H_2_O_2_, DPSCs were cultured in basal media diluted in CM and SFM at a 1:1 ratio and supplemented with an osteogenic inducer for 28 days. All groups (control, CM, and SFM) showed mineralized tissue differentiation capacity through the gene expression of odontoblast and osteoblast markers, ALP activity (ALP staining), and mineral deposition (ARS).

**Figure 5:**
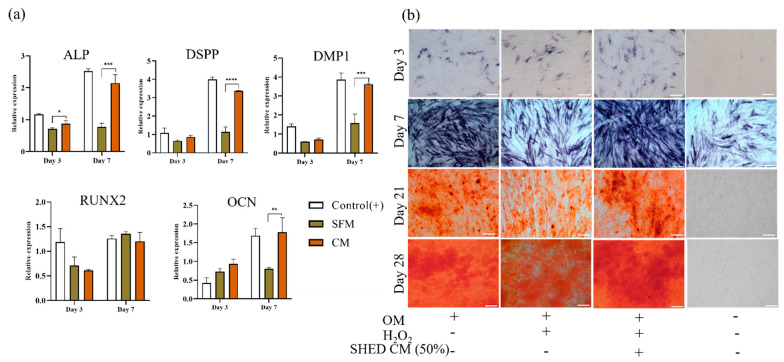
SHED CM enhanced DPSC osteogenic differentiation after exposure to 25 μM H_2_O_2_ for 24 h. (**a**) Relative gene expression, early marker at Day 3 and late marker at Day 7. (**b**) Alkaline phosphatase (ALP) and Alizarin red staining of DPSCs cultured in osteogenic differentiation media (OM) and 50% CM or SFM at Days 3, 7, 21 and 28. DPSCs cultured in growth media (αMEM, 10% FBS, 1% PS) were used as a negative control. At day 7, the upregulation of ALP activity expressed darker blue in SHED CM treatment. The red color of ARS showed stronger in SHED CM group due to the increase in the amount of mineral deposition, compared to SFM at day 21 and 28. All data are represented as the mean ± SD. The statistical significance was calculated using one-way analysis of variance (ANOVA) to compare groups. Represents * *p* < 0.05, ** *p* < 0.01, *** *p* < 0.001, **** *p* < 0.0001, ns = nonsignificant, scale bar = 200 µm.

As shown in Supplementary Figure S5a, H_2_O_2_ exposure caused the loss of osteogenic differentiation capacity. We chose a concentration of 25 μM H_2_O_2_ for further experiments. A qRT–PCR analysis was performed to evaluate the effect of SHED CM on the expression levels of key genes in bone and dentin formation, including the early marker ALP, odontoblast-specific markers DSPP and DMP1, and other osteogenic markers RUNX2 and OCN, after 3 and 7 days. According to the results shown in Figure 5a, at 3 days, most of the key genes were lower in H_2_O_2_-exposed DPSCs than in control cells, but the gene expression of OCN increased in H_2_O_2_-exposed DPSCs. However, the gene expression of H_2_O_2_-exposed DPSCs was upregulated after 7 days of CM treatment and was almost the same as that of control DPSCs (*p* > 0.05), while without CM, damaged DPSCs did not return to the normal range of gene expression, with a 2-fold increase in OCN and DMP1 and a 3-fold increase in ALP and DSPP expression (*p* < 0.001). Only RUNX2 expression was not significantly different between the groups on day 7. ALP activity and ARS confirmed the upregulation of relevant gene expression (Figure 5b). On day 3, there was no difference between the three groups in ALP staining, although ALP gene expression was higher in the control group. CM treatment promoted ALP expression on the surface of DPSCs, as ALP staining became stronger on day 7, similar to the control staining. As illustrated in Figure 5b, red-colored ARS staining indicated that calcium deposition increased on day 21.

## 4. Discussion

Intense research has recently been performed on dental pulp stem cells in regenerative medicine and tissue engineering due to their ability to self-renew, multipotency, and modulate the immune system. Dental stem cells secrete biomolecules, such as growth factors or cytokines, that positively regulate the process of tissue regeneration [23,32,34,35,36]. In the present study, we demonstrated that DPSC administration with SHED CM significantly increased cell proliferation, viability, migration, and differentiation compared with SFM in vivo. Our results agreed with other previous studies that these biological effects of SHED CM were possibly due to the high amounts of various cytokines and growth factors released during SHED culture [29,30,31,37].

The DPSCs cultured with 50% or 100% SHED CM showed increased cell proliferation after 24 h and 48 h (Figure 2a,b and Figure S3), and similar results were observed when HUVECs were incubated in SHED CM [37]. Cell-based pulp regenerative therapy involves the following two sources of stem cells: transplanted cells and host cells. The optimal microenvironment for recruiting endogenous stem cells plays a critical role in tissue regeneration. In the aged population, the repaired or regenerative pulp tissue potential is reduced, possibly due to changes in the bioenvironment and a reduction in cell migration [18,38,39]. SHED released secretomes can regulate the mobilization of DPSCs to the target tissue [29,33,40]. The effect of SHED CM on the migration of HUVECs was shown both in migration distance and wound healing area [37]. These results suggest that SHED CM promotes DPSC and endothelial cell migration into the root canal and pulp-like tissue regeneration.

Many intrinsic and extrinsic factors, such as nutrients, inflammation, mechanical stress, and oxidative stress, impact tissue regenerative capacity. Stem cells may respond differentially to different levels of oxidative stress. Under mild stress conditions, cells mainly regulate apoptosis-related gene expression, antioxidant enzyme activity, and defensive transduction pathways to fulfill antioxidative needs. However, under continuous and intense oxidative stress conditions, endogenous antioxidants may not be enough to neutralize reactive oxygen species, which cause premature aging, apoptosis, and damage to the differentiation capacity of stem cells [41,42]. Recently, much evidence has supported that proteins and peptides in MSC culture media modulate the redox microenvironment and oxidative stress, and different sources of MSC-derived CM showed differences in antioxidative capacity and a significant 10–30% increase in cell recovery [43,44,45,46]. A study by Saleem et al. revealed that CM from human BMSCs comprised antioxidant proteins that suppressed H_2_O_2-_mediated apoptosis and promoted cell proliferation [44]. Another study revealed a protective effect of MSC CM on H_2_O_2_-exposed liver cells against cell apoptosis and necrosis. The H_2_O_2_-exposed cell viability recovered by 11.5% (±2.85) when incubated with MSC CM at 24 h and increased by 15.8% (±2.79) at 48 h, respectively, by downregulating miR143 [46]. As a potential source of MSCs, SHED CM has protective properties when preventing DPSCs from H_2_O_2-_induced injury. H_2_O_2_-mediated apoptosis was lowered when 100% SHED CM was administered, with an increase of 28.09 ± 5.72% cell viability at 24 h and 55.68 ± 4.8% at 48 h compared to SFM. Although the protective effect of 50% SHED CM was not significantly different from that of SFM at 24 h of treatment (4.78 ± 5.72), an increase in cell proliferation was observed at 48 h (39.07 ± 4.8%) (Figure 3 and Figure S4b). Similarly, treatment with 50% MSC CM showed a statistically significant increase in cell viability after 7 days of incubation, but a shorter incubation time (3 days) did not induce a significant difference on Day 3. According to Jiang et al., treatment with H_2_O_2_ induces inflammation, oxidative stress, and apoptosis, and the protective effect of SHED CM on DPSCs under H_2_O_2_ may be attributed to the upregulation of the antioxidant defense of target cells through anti-inflammatory cytokines, proteins, and mRNA [47].

The differentiation to functional odontoblasts, which can secrete dentin, is one of the main objects of pulp regenerative therapy. As mentioned above, pulp regeneration in the aged population involves more challenges than in the young population because of the microenvironmental changes in the root canal and aged DPSCs [16,17,18,19,39]. Oxidative stress is well known to impair stem cell potential, including osteogenic differentiation capacity; therefore, we expected that SHED CM would restore the repaired odontoblast/osteogenic capacity of DPSCs due to H_2_O_2_-induced oxidative stress conditions [44,48,49,50]. As shown in Figure 5a, exposure to H_2_O_2_ caused changes in the expression of odonto/osteogenesis-related genes. There was a strong decrease in the expression of important markers during the odontoblast/osteogenic differentiation process, such as ALP, RUNX2, DSPP, and DMP-1, on day 3. The combination of SHED CM in OM restored the odontoblast/osteogenic differentiation capacity of H_2_O_2__-_injured DPSCs after 7 days. On day 7, the gene expression levels of ALP, RUNX-2, DSPP, and DMP-1 increased significantly compared to those in SFM. The gene expression of RUNX2 in H_2_O_2__-_treated DPSCs recovered to a level similar to that in H_2_O_2__-_untreated DPSCs. Interestingly, the gene expression of OCN increased in the H_2_O_2__-_treated groups on day 3; however, only the SHED CM-treated group showed an increase in OCN gene expression after 7 days. The level of OCN gene expression in the H_2_O_2_ untreated DPSC group also increased in the same manner as that in the SHED CM group. The increasing trend of related gene expression and ARS staining revealed significantly higher mineralize deposition in the SHED CM group than in the other groups after 21 days and 28 days (Figure 5b). In the same manner, there was an increase in mineral deposition in DPSC odontoblast/osteogenic differentiation under 100% SHED CM after 21 days and 28 days (Supplementary Figure S5b). These results indicated that SHED CM administration recovered the odontoblast/osteogenic differentiation ability of H_2_O_2__-_injured DPSCs. Our results were consistent with an in vivo study by Harika et al. that showed the enhancement of bone formation when injecting SHED CM into mouse calvaria bone defects [31]. It also reported the promotive effect of SHED CM on neovascularization, an extremely important component in tissue engineering as well as in pulp regeneration [33,37,40,43,51].

The great promise of SHED CM on tissue repair and regenerative therapies comes from multi-directional effects, rather than single effects [28,29]. SHED CM reversed acute liver injury by inducing an anti-inflammatory environment and inhibiting hepatocyte apoptosis while increasing cell proliferation and neurovascular [32]. In the same manner, treatment of osteoarthritic chondrocytes in the SHED CM improved through enhancement of anti-inflammation and an increase in extracellular matrix deposition [30]. In bone regenerative therapy, SHED CM transplantation induced more bone formation and faster bone maturation, which should be the result of osteogenesis and angiogenesis processes [31]. The pulp tissue is characterized by a highly vascularized and innervated mass of connective tissue containing various cells, such as odontoblasts and immune cells. Dentin formation is one of the most critical roles carried out by the pulp and is formed by odontoblasts [52,53]. Functional regenerated pulp tissue is required for vascular regeneration, neuronal regeneration, and dentin deposition, similar to those of natural pulp [54,55]. A recent study proved that SHED CM promoted vessel-like structure formation inside the root canal, and our study showed increased odontoblast differentiation of DPSCs in SHED CM. These results suggest that SHED CM could be a useful tool to facilitate pulp regeneration [33]. It is necessary to identify cytokines or growth factors involved in pulp-like tissue regeneration. Analyzing 310 cytokines, the result was consistent with previous studies that many well-known cytokines involved in cell proliferation and anti-apoptosis in SHED CM, such as FASLG, TIMP1, PROK1, TNFSF13B, FGF (FGF4, FGF5), and the TGF family (TGFα, TGFβ, TGFBR1) [28,29,32,34]. Our cytokine analysis strongly agreed with previous studies showing that SHED CM contained many vascular regenerative factors, including VEGF, PDGF, and bFGF [29,33,37,56]. Neuronal growth factors such as CCL2, MD1, SERPINI1, MMP-14, B2M, NRG1, and NTF3 in SHED CM may be beneficial for neuronal regeneration in pulp complex tissue [34]. SHED CM was highlighted with many inflammatory cytokines, including IL-6, IL2RA, IL17A, CCL11, CXCL1, CXCL2, CCL2IL-10, and AKT2 [30,32,34,57]. It is important that growth factors involved in odontoblast/osteogenic differentiation, such as TGF-β, BMP-4, BMP-7, FGF4, CTNNA1, CTNNB1, SCF, and CSF, are highly expressed in SHED CM [22,58]. It is suggested that the triple combined application of BMP, FGF, and VEGF significantly promoted osteogenic differentiation in a rat model study [59]. Shinji Kuroda detected that the combination of TGF-β and VEGF elevated osteogenic differentiation in both in vitro and in vivo models [60]. Similar to extracellular vesicles from other types of MSCs, such as DPSCs, SHED CM-containing cytokines were found to involve the MAPK signaling pathway, which is considered to play an important role in the growth and osteogenic differentiation of stem cells [24]. SHED CM also presented some cytokines, such as AKT2, APC, CCL11, MMP14, and NTF3, which increased the mobility of surrounding cells to root canals [29,37]. Moreover, IGFBP7 was found to enhance dental pulp stem cells’ in the growth and osteogenic/odontogenic differentiation and is released more abundantly in SHED CM [61,62]. As demonstrated in this investigation, the presence of SHED CM increased the proliferation and migration of DPSCs, counteracted H_2_O_2-_caused cell death, and recovered odontogenic/osteogenic differentiation capacity. This indicates that SHED CM can serve as an additive to improve pulp tissue regeneration; however, further in vivo experiments are needed to comprehensively understand the biological effects of SHED CM on pulp-like tissue formation.

SHED are considered an endless and excellent stem cell source, and several cell banks have been built in some countries [63]. As CM can be manufactured, freeze-dried, packaged, and transported, and its use is not required to match the donor, SHED CM has promising prospects for production as a pharmaceutical for regenerative medicine in the future [64]. The positive biological effects of SHED CM on tissue regenerative therapy were found in vitro studies but also in animal models with very appealing results [32,62,65]. To date, although SHED CM has not been tried in any clinical cases, with accumulating evidence about biological benefits and establishing a manufacturing protocol adherent with good manufacturing practice, SHED CM has potential in clinical applications. However, the effects and concentration of secreted cytokines vary from individual to individual. From different methods, it is important to standardize the concentration of SHED CM and validate its activity before commercializing CM products.

The different sources of stem cells can express distinctive cytokine profiles and properties, and their released secretomes may have different potentials for tissue regeneration [25,28]. The application of SHED CM may face challenges due to the variation in cell sources. It is suggested that further studies can be conducted on other sources of SHED to validate the biological effects of SHED CM on DPSC and determine whether SHED CM contained cytokines play critical roles in dental pulp regeneration. The appliance of SHED CM in vivo studies or clinical cases requires strategies for designs of scaffold/hydrogel incorporated or coated with CM before seeding in animal or clinical models. Due to the development of biotechnology, we expect the engineering of scaffolds/hydrogels can provide three dimensions that match the different sizes and shapes of candidates’ teeth [66,67]. When these above concerns are solved, the use of SHED CM can be more practical and bring benefits to not only pulp dental regeneration but also other tissue regeneration.

## 5. Conclusions

In this study, we showed that SHED CM was able to recover the odontogenic/osteogenic differentiation capacity of DPSCs after H_2_O_2_-induced injury. The presence of SHED CM elevated DPSC proliferation and promoted its migration, while reduced H_2_O_2_ caused cell apoptosis. These results were followed by the presence of a variety of growth factors and cytokines in SHED CM. Therefore, SHED CM can serve as an additive to improve the success of pulp tissue regenerative therapy. In vitro, the role of SHED CM was demonstrated compared to SFM, and further in vivo studies should be conducted to evaluate the efficiency leading to the formation of functional pulp-like tissue inside the root canal.

**Figure 6:**
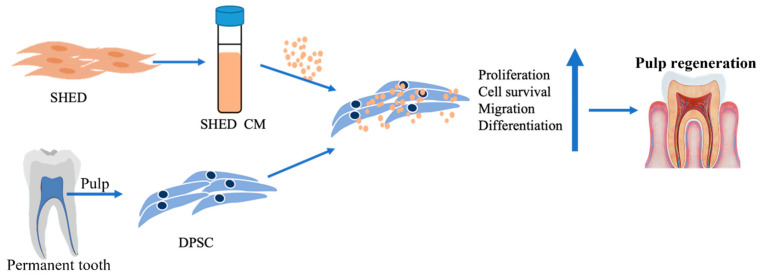
Schematic diagram of the effect of SHED CM on DPSC culture and odontoblast differentiation. SHED CM increased cell proliferation and progenitor cell recruitment, protected cells from oxidative stress, and enhanced cell differentiation.

Our study expands the knowledge of SHED-derived cytokines and their biological effects on tissue regenerative therapy, especially on DPSCs in pulp regenerative therapy (Figure 6).

## Supplementary Materials

The following are available online at https://www.mdpi.com/article/10.3390/biomedicines10040906/s1, Figure S1: (a) Primary culture of DPSCs. Cell adherence could be observed on the culture flask wall 16–24 h after seeding, and clustered SHED was observed on Day 4 or 6. Most cells had a typical fibroblast-like appearance. (b) Primary culture of SHED and multiple lineage differentiation capacity of SHED was demonstrated by bone mineralization (ARS) and neutral lipid accumulation (oil Red O). Scale bar = 200 µm, Figure S2. Illustration of SHED CM collection. SHED passage #5 was cultured to 70%–80% confluence and replenished with serum-free αMEM. After 48 h of incubation at 37 °C in 5% CO_2_, the conditioned medium (CM) was collected and centrifuged at 3000 rpm for 5 minutes at 4 °C to remove cell debris, and then the supernatant was passed through 0.2 μm filters to obtain the final CM. Control-conditioned media was collected from serum-free αMEM (SFM) and incubated for 48 h under the same conditions as conditioned medium. The number of live cells was also calculated to ensure the cell viability was higher than 90%. All CM and SFM were subpackaged in 2 mL tubes and stored at −80 °C, Figure S3. Effect of SHED CM on DPSC proliferation and migration. (a) Presentative Live & Dead images of SHED 48 h after treatment with 100% CM versus 50% CM and SFM. The scale bar represents 200 µm. (b) The effect of SHED CM after treatment for 48 h on the proliferation of DPSCs was quantified by CCK-8 assay. n = 6, Figure S4. (a) SHED exposure to different concentrations of H_2_O_2_. DPSCs were cultured with 100, 200, 300 and 400 µM H_2_O_2_ for 12 h. Representative live & dead images show the H_2_O_2_ induced oxidative stress effect on SHED. Cell viability was determined by CCK-8 analysis. (b) DPSCs were cultured with 200 µM H_2_O_2_ for 12 h before shifting to CM, 50% CM and SFM for another 36 h. Representative live & dead images of DPSCs. Cell viability was determined by CCK-8 analysis. All data are represented as the mean ± SEM. The statistical significance was calculated using one-way analysis of variance (ANOVA) to compare groups. Represents **** *p* < 0.0001, ns=not significant, scale bar = 200 µm, Figure S5. (a) DPSC osteogenic differentiation after 24 h of exposure to different H_2_O_2_ concentrations, representative bright field images and ARS staining. (b) Treatment with 100% SHED CM enhanced DPSC osteogenic differentiation after exposure to 25 M H_2_O_2_ for 24 h and ARS staining at Days 21 and 28. Scale bar = 200 µm, Table S1. Biological functions of the top 50 cytokines in SHED CM (Gene Ontology database)
