# Supplementary material for: Investigating the Effects of Conditioned Media from Stem Cells of Human Exfoliated Deciduous Teeth on Dental Pulp Stem Cells
Source: Biomedicines. 2022 Apr 15;10(4):906. doi: 10.3390/biomedicines10040906 (PMC9027398; doi:10.3390/biomedicines10040906)
Supplement: Supplementary file 1 [file biomedicines-10-00906-s001.zip › biomedicines-1669166-supplementary.pdf]

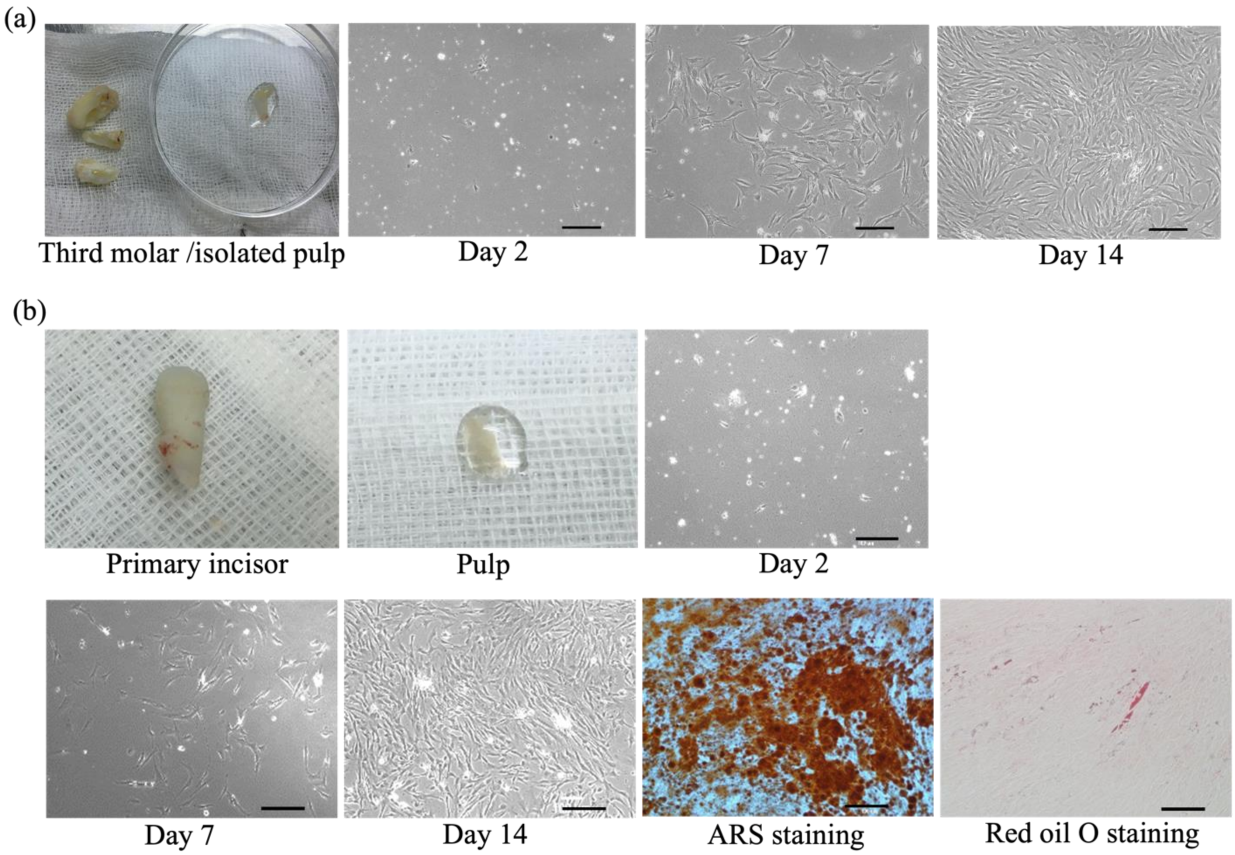

Supplemental Figure S1. (a) Primary culture of DPSCs. Cell adherence could be observed on the culture flask wall 16-24 hours after seeding, and clustered SHED was observed on Day 4 or 6. Most cells had a typical fibroblast-like appearance. (b) Primary culture of SHED and multiple lineage differentiation capacity of SHED was demonstrated by bone mineralization (ARS) and neutral lipid accumulation (oil Red O). Scale bar = 200 µm.

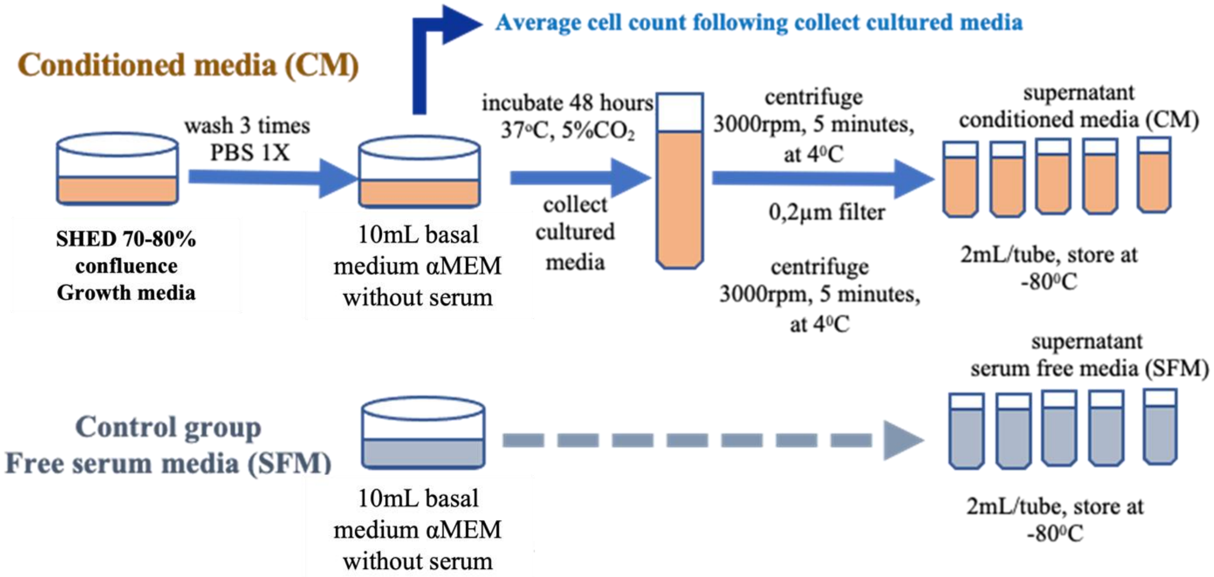

Supplemental Figure S2. Illustration of SHED CM collection. SHED passage #5 was cultured to 70%–

80% confluence and replenished with serum-free **α**MEM. After 48 hours of incubation at 37 °C in 5% CO_2, the conditioned medium (CM) was collected and centrifuged at 3000 rpm for 5 minutes at 4 °C to remove cell debris, and then the supernatant was passed through 0.2 μm filters to obtain the final CM. Control-conditioned media was collected from serum-free αMEM (SFM) and incubated for 48 hours under the same conditions as conditioned medium. The number of live cells was also calculated to ensure the cell viability was higher than 90%. All CM and SFM were subpackaged in 2 mL tubes and stored at −80 °C.

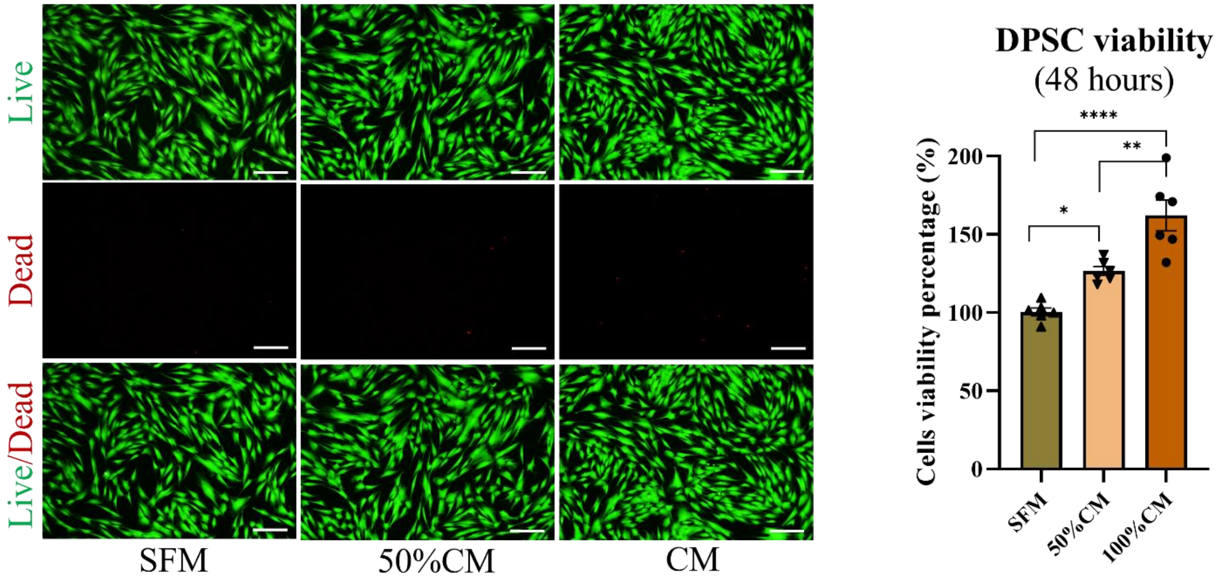

Supplemental Figure S3. Effect of SHED CM on DPSC proliferation and migration. (a) Presentative Live & Dead images of SHED 48 h after treatment with 100% CM versus 50% CM and SFM. The scale bar represents 200 μm. (b) The effect of SHED CM after treatment for 48 h on the proliferation of DPSCs was quantified by CCK-8 assay. n=6

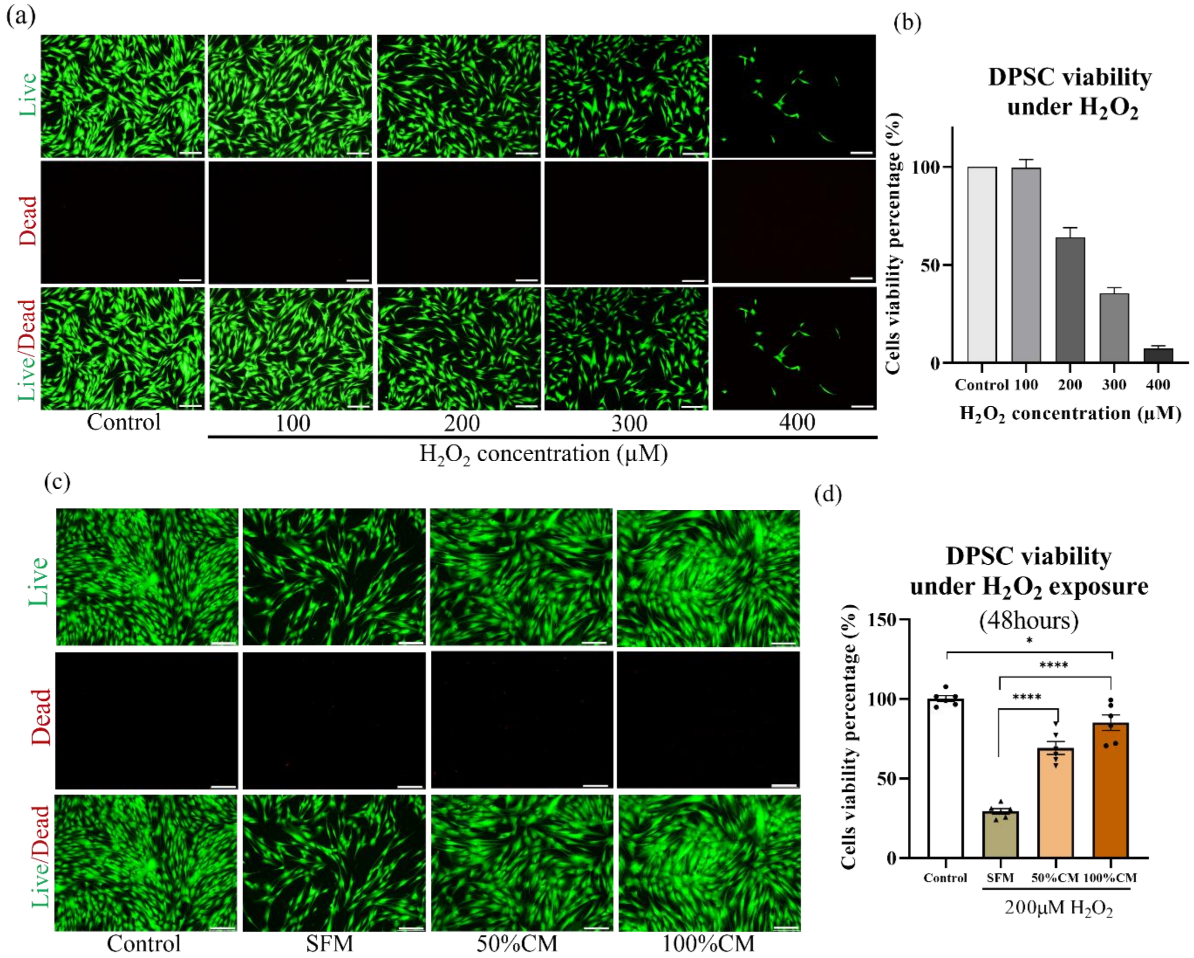

Supplemental Figure S4. (a) SHED exposure to different concentrations of H_2O_2. DPSCs were cultured with 100, 200, 300 and 400 μM H_2O_2 for 12 hours. Representative live & dead images show the H_2O_2 induced oxidative stress effect on SHED. Cell viability was determined by CCK-8 analysis. (b) DPSCs were cultured with 200 μM H_2O_2 for 12 hours before shifting to CM, 50% CM and SFM for another 36 hours. Representative live & dead images of DPSCs. Cell viability was determined by CCK-8 analysis. All data are represented as the mean ± SEM. The statistical significance was calculated using one-way analysis of variance (ANOVA) to compare groups. Represents **** $p < 0.0001$, ns=not significant, scale bar = 200 μm.

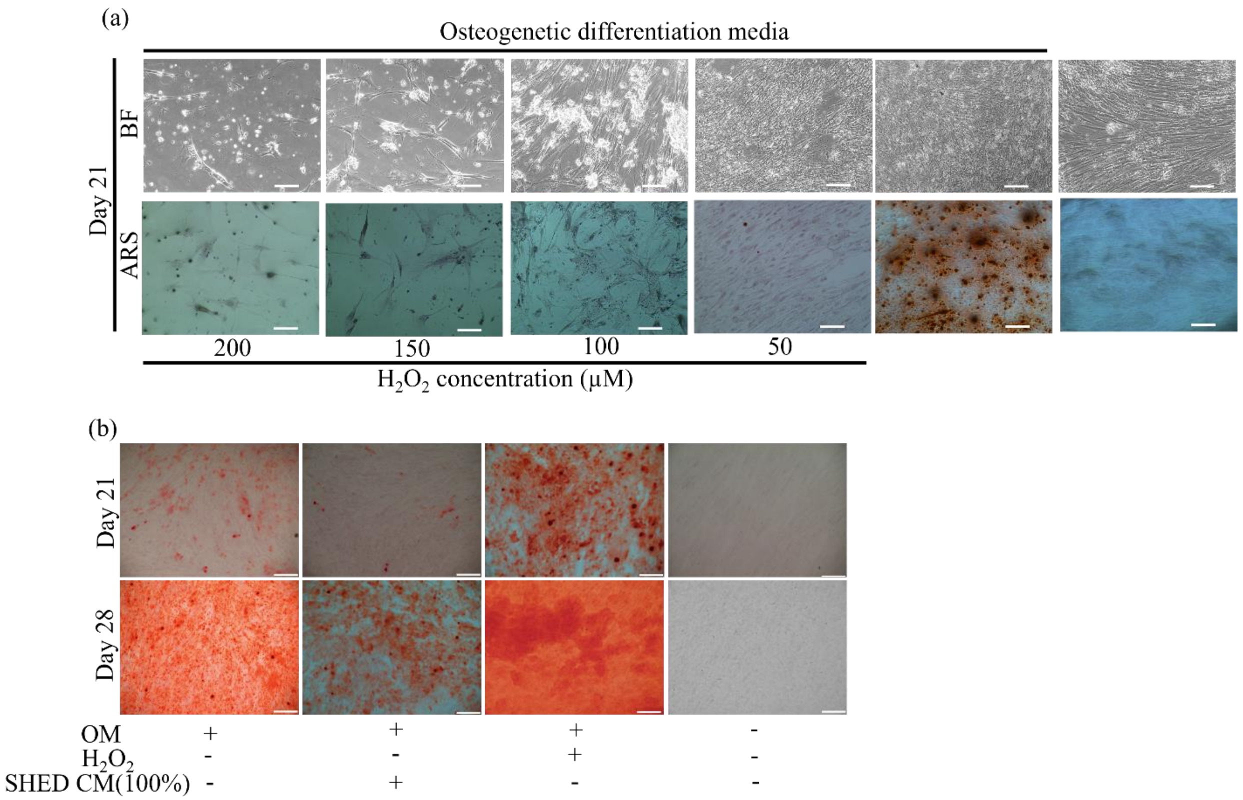

Supplemental Figure S5. (a) DPSC osteogenic differentiation after 24 hours of exposure to different H_2O_2 concentrations, representative bright field images and ARS staining. (b) Treatment with 100% SHED CM enhanced DPSC osteogenic differentiation after exposure to 25 μM H_2O_2 for 24 hours and ARS staining at Days 21 and 28. Scale bar = 200 μm.

Supplemental Table S1. Biological functions of the top 50 cytokines in SHED CM (Gene Ontology database)

**N**	**Gene Symbol**	**Protein names**	**Biological functionals (DAVID annotation)**
1	ADIPOQ	Adiponectin	Proliferation, survival
2	AKT2	RAC-beta serine/threonine-protein kinase	Proliferation, migration
3	APC	Adenomatous polyposis coli protein	Migration
4	ARTN	Artemin	Proliferation, survival
5	B2M	Beta-2-microglobulin	Survival
6	BMP4	Bone morphogenetic protein 4	Proliferation, survival, migration, odontoblast/osteogenic differentiation
7	BMP7	Bone morphogenetic protein 7	Odontoblast/osteogenic differentiation
8	CCL11	C-C motif chemokine 11	Survival, migration
9	CCL19	C-C motif chemokine 19 chemokine	Proliferation, survival, migration
10	CCL2	C-C motif chemokine 2 factor	Proliferation, survival, migration
11	CTNNA1	Catenin alpha-1	Odontoblast/osteogenic differentiation
12	CTNNB1	Catenin beta-1	Odontoblast/osteogenic differentiation
13	CXCL1	Growth-regulated alpha protein	Migration
14	ERBB2	Receptor tyrosine-protein kinase erbB-2	Proliferation, survival, migration, odontoblast/osteogenic differentiation

15	ERBB3	Receptor tyrosine-protein kinase erbB-3	Proliferation, survival, migration, odontoblast/osteogenic differentiation
16	ERCC6	DNA excision repair protein ERCC-6	Odontoblast/osteogenic differentiation
17	FASLG	Tumor necrosis factor ligand superfamily member 6	Inflammation, apoptosis
18	FGF4	Fibroblast growth Factor 4	Proliferation, survival, odontoblast/osteogenic differentiation
19	FGF5	Fibroblast growth Factor 5	Proliferation, survival, odontoblast/osteogenic differentiation
20	HDAC9	Histone deacetylase 9	Inflammation, apoptosis
21	IGFBP7	Insulin-like growth factor-binding protein 7	Proliferation, survival
22	IL2RA	Interleukin-2 receptor subunit alpha	Proliferation, survival
23	ITGB5	Integrin beta-5	Proliferation
24	LGALS3	Galectin-3	Migration
25	MDK	Midkine	Proliferation, migration
26	MMP1	Matrix metalloproteinase 1	Proliferation
27	MMP10	Matrix metalloproteinase-10	Proliferation
28	MMP11	Matrix metalloproteinase-11	Proliferation
29	MMP13	(Matrix metalloproteinase-13	Proliferation, odontoblast/osteogenic differentiation
30	MMP14	Matrix metalloproteinase-14	Proliferation, migration odontoblast/osteogenic differentiation
31	MMP15	Matrix metalloproteinase-15	Proliferation
32	MMP16	Matrix metalloproteinase-16	Proliferation, odontoblast/osteogenic differentiation
33	MMP8	Matrix metalloproteinase 8	Proliferation, odontoblast/osteogenic differentiation
34	NANOG	Homeobox protein NANOG	Proliferation, survival
35	NRG1	Pro-neuregulin-1	Proliferation, migration odontoblast/osteogenic differentiation
36	NTF3	Neurotrophin-3	Migration, survival
37	PROK1	Prokineticin-1	Proliferation
38	S100A1	Protein S100-A1	Angiogenesis
39	SELE	E-selectin	Migration, inflammation
40	SERPINI1	Neuroserpin	Neurogenesis
41	STAT1	Signal transducer and activator of transcription 1-alpha/beta	Inflammation, apoptosis
42	STAT5A	Signal transducer and activator of transcription 5A	Inflammation
43	TGFA	Protransforming growth factor alpha	Proliferation, survival, migration
44	TGFB1	Transforming growth factor beta-1	Proliferation, survival, migration, odontoblast/osteogenic differentiation
45	TGFBR2	Transforming growth factor-beta receptor type-2 receptor type II	Survival, migration, odontoblast/osteogenic differentiation
46	TIMP1	Tissue inhibitor of metalloproteinases 1	Proliferation, survival

47	TNFSF13B	Tumor necrosis factor ligand superfamily member 13B	Inflammation
48	TNFSF15	Tumor necrosis factor ligand superfamily member 15 growth inhibitor	Apoptosis
49	TNFSF18	Tumor necrosis factor ligand superfamily member 18	Survival, inflammation
50	VEGFB	Vascular endothelial growth Factor B	Proliferation, survival, migration
